# Comparative primate analysis shows that humans are not unique in having a tight cephalopelvic fit at birth

**DOI:** 10.1038/s41559-026-03102-5

**Published:** 2026-06-29

**Authors:** Nicole Torres-Tamayo, Stefan Schlager, Eishi Hirasaki, Tim D. Smith, Todd C. Rae, Lia Betti

**Affiliations:** 1 Department of Anthropology, University College London, London, UK; 2 Institute of Evolutionary Medicine, University of Zurich, Zurich, Switzerland; 3 Hominin Origins Research Group, Institut Català de Paleontologia Miquel Crusafont, Universitat Autònoma de Barcelona, Cerdanyola del Vallés, Spain; 4 Department of Oral and Maxillofacial Surgery, University Medical Center Freiburg, Freiburg im Breisgau, Germany; 5 Institute for the Evolutionary Origins of Human Behavior, Kyoto University, Inuyama, Japan; 6 Department of Health and Rehabilitation Sciences, Program of Physical Therapy, Slippery Rock University, Slippery Rock, PA USA; 7 Department of Ecology and Evolution, School of Life Sciences, University of Sussex, Falmer, UK

**Keywords:** Biological anthropology, Evolutionary theory

## Abstract

Human childbirth is regarded as uniquely difficult among primates, due to a tight cephalopelvic fit thought to result from an evolutionary trade-off between adaptations to bipedal locomotion and increasing brain size. This impression, however, may be an artefact of past adoption of anthropocentric measurements that underestimate birth challenges in non-human primates. Here we re-evaluate cephalopelvic proportions using species-specific three-dimensional data of the pelvic inlet along with neonatal cranial dimensions from a broad sample of extant primates. Results reveal that maternal body size is a key factor to consider. A tight cephalopelvic fit occurs in species with proportionately larger neonates, smaller pelves or a combination of both. The latter is the case in humans, producing the tightest fit among extant apes, but a similar combination of factors explains much more extreme cephalopelvic proportions in other species. Our findings reveal a diversity of obstetrical dilemmas across primates.

## Abstract (web summary)

Analysing cephalopelvic proportions among 29 extant primate species, the authors show that a tight cephalopelvic fit is not unique to humans, with comparable or tighter fits occurring in several non-human primate species, including macaques, squirrel monkeys and bushbabies.

## Main

In the 1940s, the primatologist Adolph H. Schultz conducted the first comparative investigations of cephalopelvic proportions (CPP) across primates, estimating the fit of the neonatal head into the superior aperture of the maternal birth canal (more specifically, the pelvic inlet) for a few taxa^1^. Using standard anthropometric techniques developed on humans, the results suggested that the birth canal inlets of non-human great apes are more capacious relative to neonatal size than that of humans and have a markedly more oval shape, elongated sagittally (anterior–posteriorly). This interpretation, and in particular the widely reproduced figure, was highly influential on subsequent work^2,3^, effectively defining anthropological conceptualization of birth across primates, and reinforcing traditional assumptions that (1) human childbirth is uniquely difficult due to a tight cephalopelvic fit and a distinct pelvic canal shape, and (2) other primates face little to no birth obstruction^4–7^(though he noted a tight cephalopelvic fit in some monkeys). Later studies, accepting these assumptions without re-examination, sought to identify the evolutionary and anatomical factors that make human parturition particularly challenging. Washburn proposed that humans experienced a unique evolutionary trade-off between a larger brain and a more compact bony birth canal evolved for bipedalism^8^. This ‘obstetrical dilemma’ was interpreted subsequently as leading to a notably tight fit between a large fetal head and an unusually shaped birth canal^9,10^ as well as complex, rotational delivery patterns^6^. Washburn hypothesized that the key human adaptation to keep CPP manageable has been secondary altriciality, where a larger portion of brain growth occurs after birth instead of in utero, limiting neonatal head size and resulting in highly dependent infants^8^.

Behavioural evidence, however, indicates that parturition can also be a complex and occasionally problematic process in non-human primates, potentially due to high CPP in some species^11^. Cases of obstructed labour, prolonged delivery and stillbirth have been documented in both wild and captive populations across several species, including other great apes^12^, squirrel monkeys^13–15^, macaques^16^, wild mandrills^17^, baboons^18^ and vervet monkeys^19^. In a Saimiri captive colony, 16% of infants were reported to be stillborn and 34% died within 100 days of birth^20^. Some non-human primates perform postural adjustments during labour and there are reports of assistance from conspecifics to manage birth-related challenges^7,21,22^. These findings challenge the assumption that parturition in other primates is easy in comparison to human birth and highlight the need for a broader, comparative framework to understand the evolution of obstetric constraints.

**Fig. 1:**
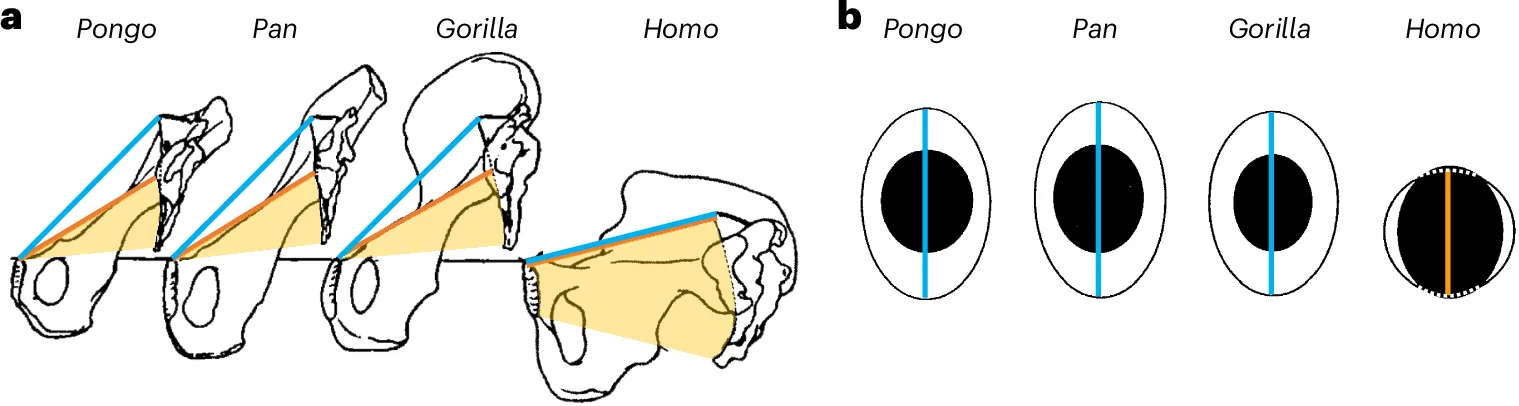
Comparative orientation of the pelvic inlet and cephalopelvic constraints in great apes and humans. **a**, Comparison between the traditional anterior–posterior inlet diameter measured between the sacral promontory and the top of the pubic symphysis (blue lines), which assumes this is the most constraining axis in all primates, and more realistic obstetrically relevant diameters and space (orange lines and shaded area). Pelves of different primates are represented in sagittal section and aligned with an approximately vertical pubic symphysis. Note how the entirety of the sacrum contributes to delimiting the canal passage in humans. **b**, Schematic depiction of cephalopelvic fit at the inlet in each species, showing neonatal head (filled oval) relative to inlet shape and orientation (open oval). The diameters illustrated correspond to those shown in **a**. Illustrations redrawn with permission from: **a**, ref. ^1^, John Wiley and Sons; **b**, ref. ^57^, John Wiley and Sons.

The discrepancy between anatomical observations and reported birth difficulties in non-human primates may lie in the methodological protocols used in earlier comparative anatomy studies. The methods developed by Schultz^1^ and later adopted by others^2,3,23^ have been questioned^24,25^. By applying standard human obstetric measurements to non-human primate anatomy, pelvic inlets are treated as idealized, generic ovals (Fig. 1). The anterior–posterior measurement used to define the dimension of the birth canal inlet is based on a key human obstetrical diameter—the distance between the upper part of the pubic symphysis and the sacral promontorium (Fig. 1a). Although this distance is often the most limited space the human neonate head must traverse in that orientation, therefore representing an important human obstetric constraint, this is actually not the case in non-human primates. In non-human primates, the sacrum is positioned higher relative to the pubis, meaning that the superior sacral margin does not directly constrain the space available for the neonate; instead, the smallest sagittal canal diameter occurs toward the inferior portion of the sacrum^24,25^ (Fig. 1b). Humans are an exception in this regard, as selection for a shortened lower ilium and a reduced distance between the acetabulum and sacroiliac joints^26^, together with spinopelvic adaptations for bipedal locomotion, has shifted the constraining plane toward the top of the sacrum^27^. Another important point is that neonatal head diameters have been based previously on sinciput presentation (presenting the top of the head, from the forehead to the occipital protuberance, also called military presentation), which does not reflect common head orientation during birth in either humans or other primates^20^. In other words, the measurements adopted do not correspond to the most relevant pelvic and head dimensions for parturition across species, misrepresenting cephalopelvic relationships in non-human primates and providing an unreliable basis for understanding the apparent uniqueness in human childbirth.

Here we move beyond an anthropocentric framework and adopt a wider zoological approach to evaluate cephalopelvic fit across a broad comparative sample of extant primates, emphasizing the use of functionally appropriate, species-specific measurements rather than relying on human-specific dimensions that may not capture obstetrically relevant constraints across taxa. Species-specific pelvic morphology and variation in fetal head positioning during birth are used to more accurately reconstruct how feto–pelvic relationships vary across primates and clarify where humans sit within this broader pattern. We focus on the pelvic inlet to ensure a valid comparison with Schultz’s widely cited work and other previous evaluations. We quantify the size and shape of the obstetrically relevant (constraining) birth canal inlet using three-dimensional (3D) morphometric analysis of landmarks and curve semi-landmarks on the bony pelvis. To replace the traditional plane used by Schultz, we employ a new repeatable osteological plane for the pelvic inlet that provides a more anatomically realistic estimate of inlet geometry, better reflecting the contribution of the sacrum to the bony ring that the neonate needs to traverse (Methods). Similar positioning of the intersection between the sacrum and the inlet was recorded by ref. ^20^, whose radiographs during labour allowed direct measurement of fetal position and maternal and fetal head dimensions during birth in baboons and squirrel monkeys. Recent 3D evaluations of the primate birth canal have suggested that the most restricted part of the canal in some species occurs, in fact, at a lower level, involving the bottom end of the sacrum^11,24^. This may indeed be the case in several species, but the contribution of sacral nutation—defined as the change in sacral inclination relative to the iliac bones, facilitated by the elasticity of the connective tissues at the sacroiliac joint—to the space available to the fetus at this lower level remains unclear. During parturition, sacral nutation is thought to increase the anterior–posterior diameter of the canal toward the distal sacrum, at least in humans^28^. Unfortunately, comparative data on soft-tissue flexibility and the magnitude of sacral nutation in other primate species are lacking. This is an additional reason why our analyses are restricted to the inlet, where the pelvic bones form a more tightly articulated skeletal ring that fully surrounds the neonate during birth, providing a more rigid and comparable structure across taxa.

In humans, birth usually occurs in occiput anterior presentation, where the back of the fetal head (occiput) leads, and cranial diameters are minimal. In non-human primates, face presentations are more common and also present smaller diameters than sinciput presentations^20^. Thus, the traditional measurement of fetal head breadth and length in sinciput presentation is not representative of birth constraints in either group. More realistic estimates of cephalopelvic fit are obtained by combining head breadth with head height in face (submento–bregmatic diameter, non-human) or occiput presentation (suboccipito–bregmatic diameter, humans). We fit simplified neonatal head models virtually in both presentations into maternal pelvic inlets to assess cephalopelvic fit across species. Moving away from standard anthropocentric measurements and adopting a species-specific approach, we reassess current assumptions about the difficulty of birth in humans relative to other primates.

## Results

**Fig. 2:**
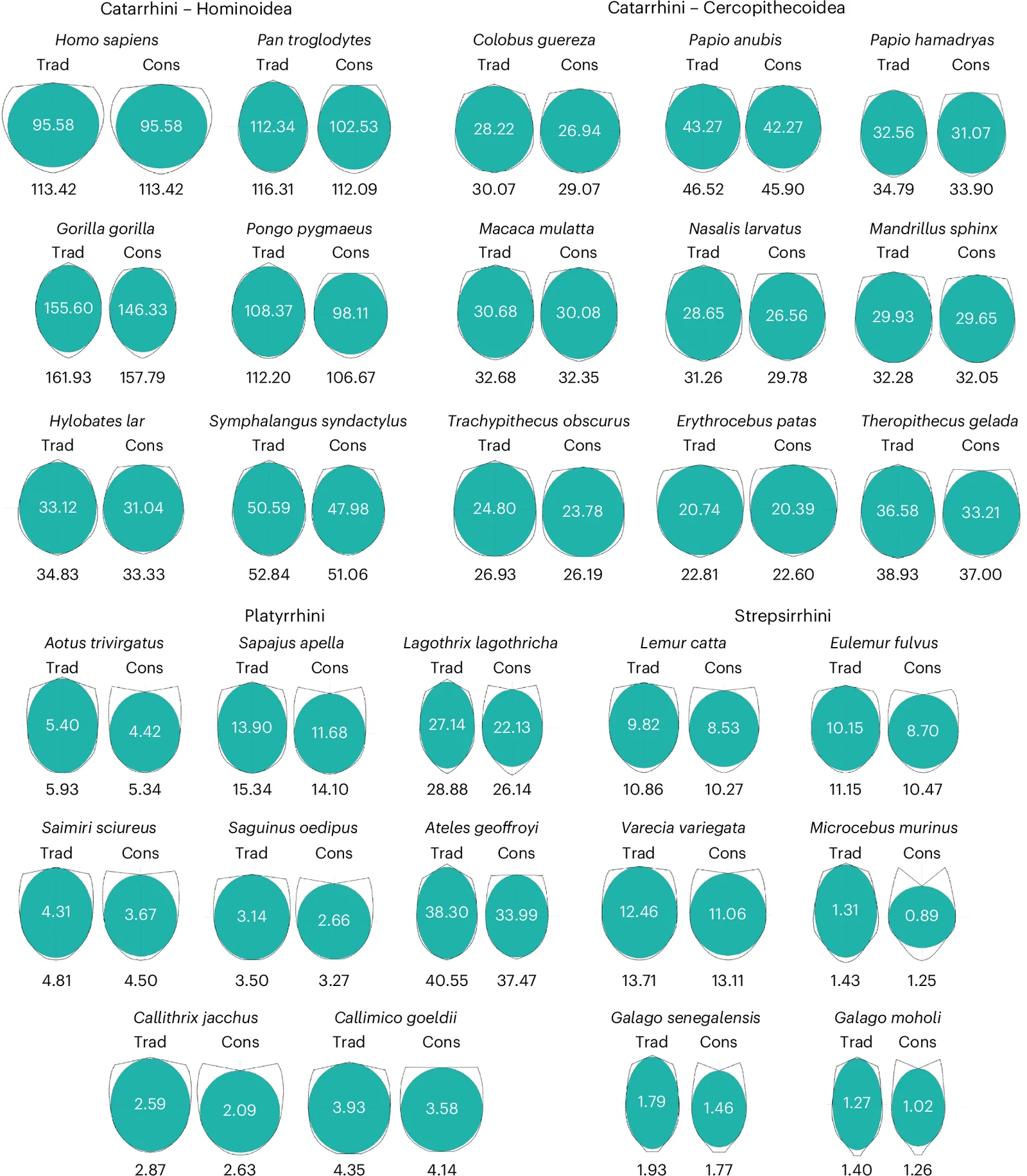
Traditional versus species-specific measurements of the pelvic inlet area in humans and non-human primates. Comparison of pelvic inlet shape and area using traditional (Trad) human-based obstetric measurements, and the obstetrically constraining (Cons) inlet based on species-specific 3D geometric morphometric measurements for each species, with overall inlet area values (cm^2^) reported at the bottom of the polygons. The grey polygons represent the reconstructed outlines of the bony birth canal based on pelvic landmarks and semi-landmarks, calculated from the species mean birth canal shape for each taxon. Green ellipses represent the maximum elliptical area that could be theoretically occupied by the neonatal head, without accounting for physiological limits to cranial deformation, with area values reported in the centre. In all non-human primates, the new approach returns a smaller and less sagittally elongated inlet, resulting in reduced available space for fetal passage.

Our results challenge the traditional perception of a uniquely tight cephalopelvic fit at human birth by showing more extreme CPP in several non-human primate species and a tighter fit in more taxa than previously recognized. In all non-human primate species evaluated, the obstetrically relevant area of the pelvic inlet decreases when we use a species-specific approach, with respect to previous anthropocentric measurements, and the obstetrically constraining inlet is characterized as rounder (less sagittally elongated) (Fig. 2). Accordingly, the portion of that area that could realistically be filled by the neonate’s head also decreases in all species but humans (Fig. 2 and Supplementary Table 1).

The obstetrically constraining inlets (compared with traditional models based on human standards), are smaller than previous reconstructions by an average of 11% in non-human primates, reaching more than 18% reduction in some species (for example *Aotus trivirgatus*, *Galago moholi*, *Galago senegalensis* and *Lagothrix lagothricha*). A paired *t*-test confirmed a significant difference between the inscribed ellipse areas for the traditional and constraining inlets (*t* = 3.65, df = 31, *P* < 0.001).

**Fig. 3:**
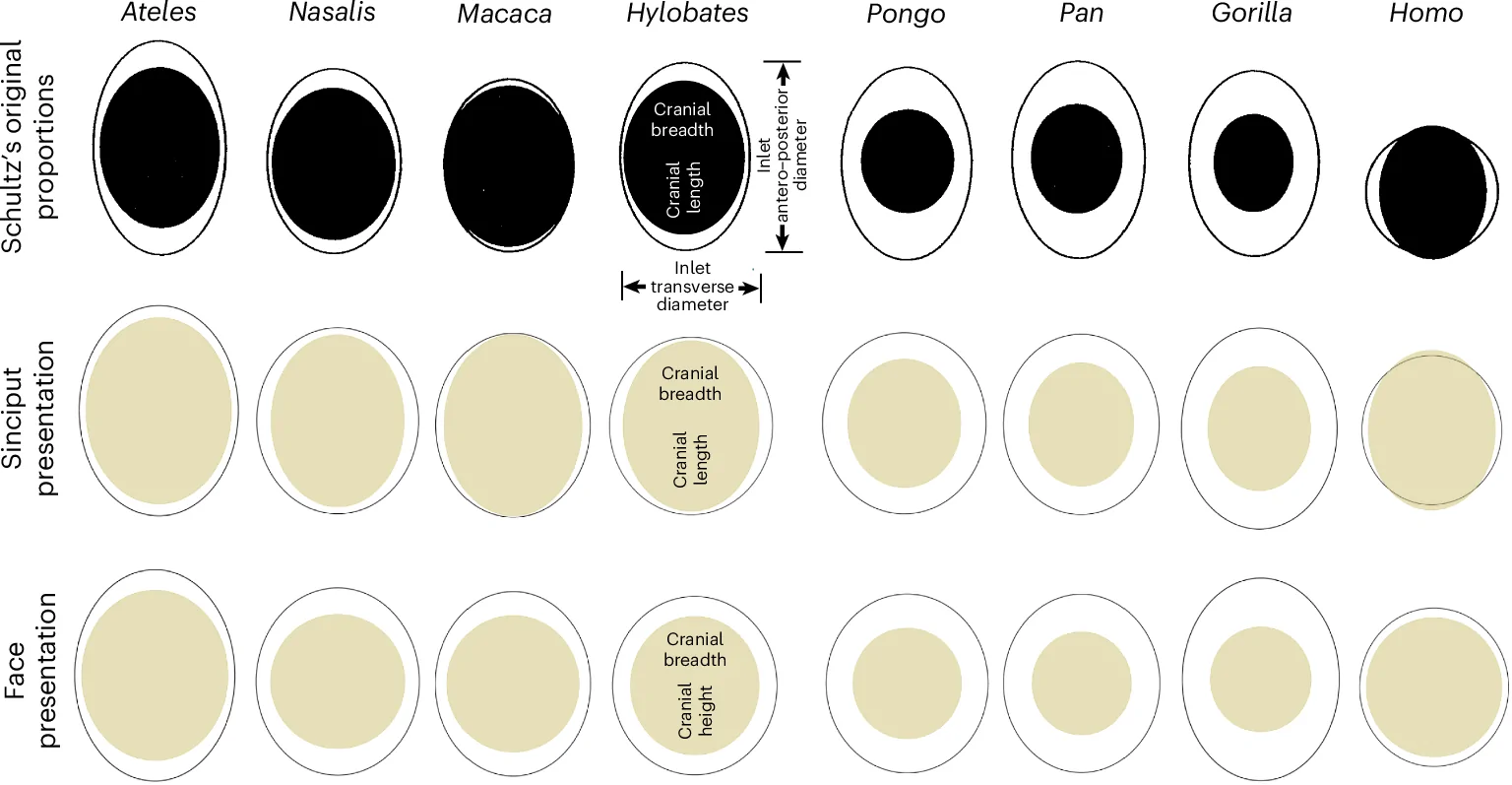
CPP across eight primate genera based on traditional and updated inlet and head size reconstructions. Top row: Reproduction of schematic diagrams from ref. ^57^ showing cephalopelvic fit based on the measurements by Schultz^1^, with neonatal cranial dimensions (black ellipses) plotted against idealized pelvic inlets (open ellipses), assuming sinciput presentation. Middle row: Updated reconstructions using species-specific 3D geometric morphometrics of the pelvic inlet to estimate elliptical available space, and neonatal head dimensions in sinciput presentation. Bottom row: Equivalent reconstructions in face/occiput presentation, where the vertical axis of the neonatal ellipse reflects cranial height rather than length. Beige ellipses represent neonatal dimensions; open ellipses represent the space available in the obstetrically constraining maternal pelvic inlet. These reconstructions show that a face/occiput presentation notably alleviates the tight fit between neonate head and birth canal, especially in humans and monkeys. Note that great apes exhibit a relatively ample inlet in both presentations. Illustration adapted with permission from ref. ^57^, John Wiley and Sons.

To compare our results with previous studies, we recreated Schultz’s cephalopelvic fit diagram^1^ that is today replicated in textbooks of human evolution, anatomy, midwifery and obstetrics (Fig. 3). We compared the maximum ellipse that can be fitted into the obstetrically constraining plane of the maternal birth canal inlet (representing the space available for the neonate’s head) with a projection of the neonate head in sinciput and face/occiput presentation. Because in humans neonatal cranial height is similar in face and occiput presentation, we combine human occiput presentation with other primates’ face presentation in a single category (face/occiput presentation) in our results. Schultz’s original representations suggest that cephalopelvic fit is notably tight in *Homo*, with ample space in most non-human primates, particularly in great apes, and highlights a very elongated inlet in non-human primate species with respect to humans. Our reconstructions, using species-specific 3D morphometrics, produce smaller and less sagittally elongated maternal inlets in non-human species, although confirming a broadly similar pattern of fit in Schultz’s head (sinciput) presentation. In the more realistic face/occiput presentation, however, the height of the head (instead of the length) is combined with head breadth to generate a neonatal head ellipse area on average 23.6% smaller than in sinciput presentation (Supplementary Table 1), resulting in a less constrained cephalopelvic fit in most species (Fig. 3).

**Fig. 4:**
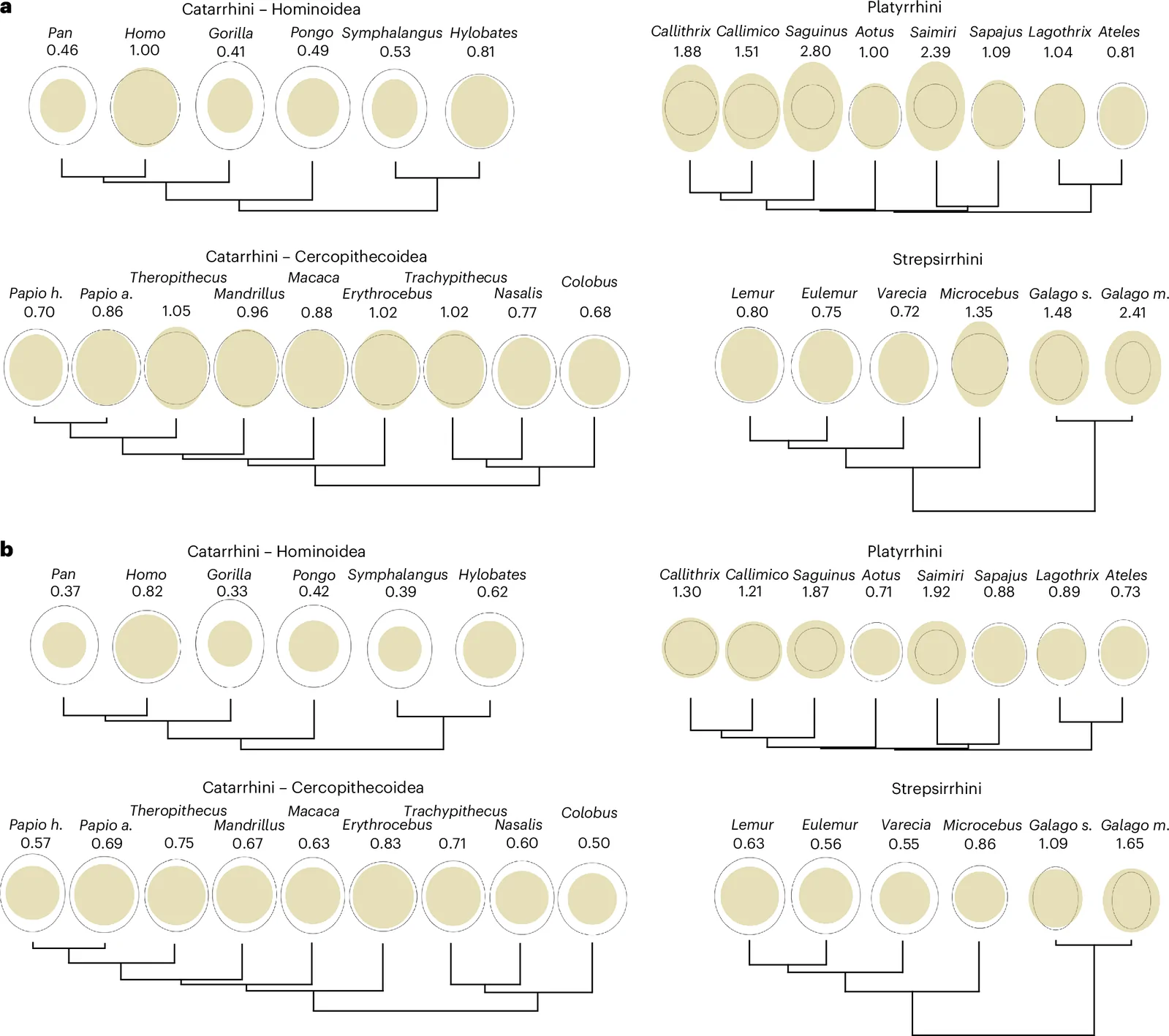
Phylogenetic distribution of CPP in primates using species-specific measurements of the inlet and assuming neonatal sinciput or face/occiput presentation. **a**,**b**, Cephalopelvic fit visualized at the obstetrically constraining plane of the pelvic inlet across a wide sample of primates, shown in sinciput (**a**) and face/occiput (**b**) presentation. For each species, neonatal cranial dimensions (beige ellipses) are plotted within maternal pelvic inlet space (open ellipses), highlighting relative fit, and actual CPP values are shown below each genus name. Taxa are grouped into three main clades: Catarrhini (Hominoidea and Cercopithecoidea), Platyrrhini and Strepsirrhini, and arranged according to within-clade phylogenetic relationships.

When extended to other primate species and mapped onto cladograms (Fig. 4), high CPP are clearly not restricted to any one clade. Within apes, our species exhibits the tightest fit, with the fetal head nearly filling the inlet. Lar gibbons (*Hylobates*) also show a moderate constraint, whereas other apes retain relatively spacious birth canals. Tight fits are also present in several cercopithecoids (macaques, mandrills, geladas). In American monkeys (Platyrrhini), particularly high CPP are seen in the small-bodied taxa callitrichids and squirrel monkeys, where the fetal head is actually larger than the birth canal. Strepsirrhines show similar variation, with bushbabies (for example, *Galago*) exhibiting particularly disproportionate fits. Modelling CPP with face presentation consistently reduces cephalopelvic mismatch, particularly in platyrrhines and strepsirrhines, suggesting that face-first fetal orientation may serve as a compensatory mechanism to avoid obstructed labour. While humans remain among the most constrained species of apes, the even tighter fits in several primates, such as galagos, squirrel monkeys and patas monkeys, indicate that birth constraints are not unique to our evolutionary lineage. This raises the possibility that tight CPP represent the ancestral condition in crown primates, with looser fits arising later in some lineages.

**Fig. 5:**
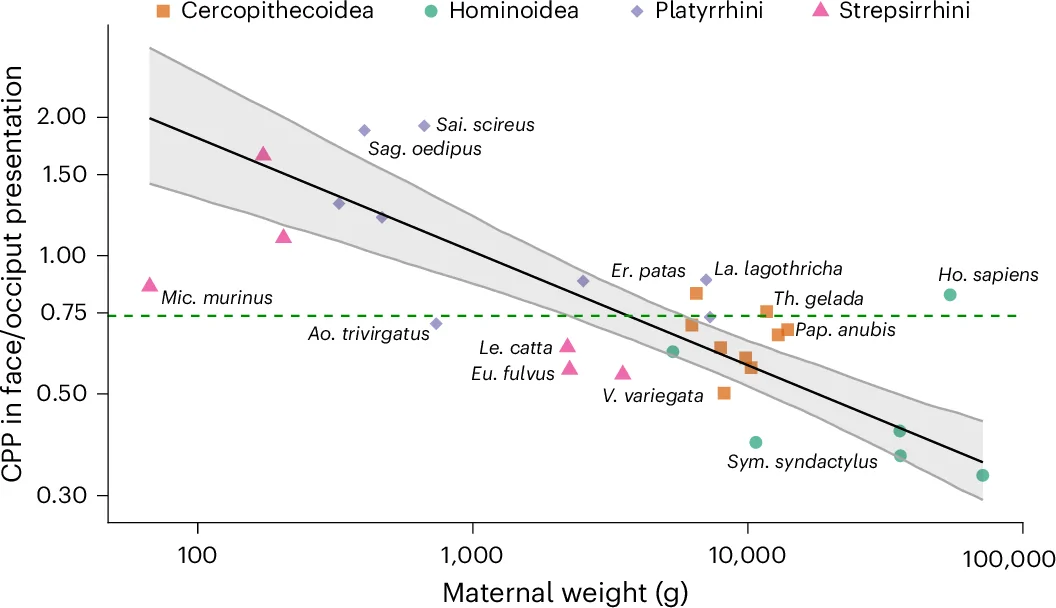
Relationship between CPP and maternal body size across primates. Regression on log scale of CPP (in face/occiput presentation) against maternal weight across 29 primate species. The black line and shading reflect the allometric relationship line and 95% confidence interval (CI), estimated through SMA regression (slope = −0.247, 95% CI −0.322 to −0.190, intercept = 0.750, *R*^2^ = 0.538, *P* < 0.0001). The slope is significantly different from what is expected under isometry (isometry slope = 0, *P* < 0.0001), represented by a dashed green line. CPP decreases significantly with increasing maternal size, reflecting allometric scaling. Species that deviate the most from the fitted line have been labelled.

**Extended Data Fig. 1:**
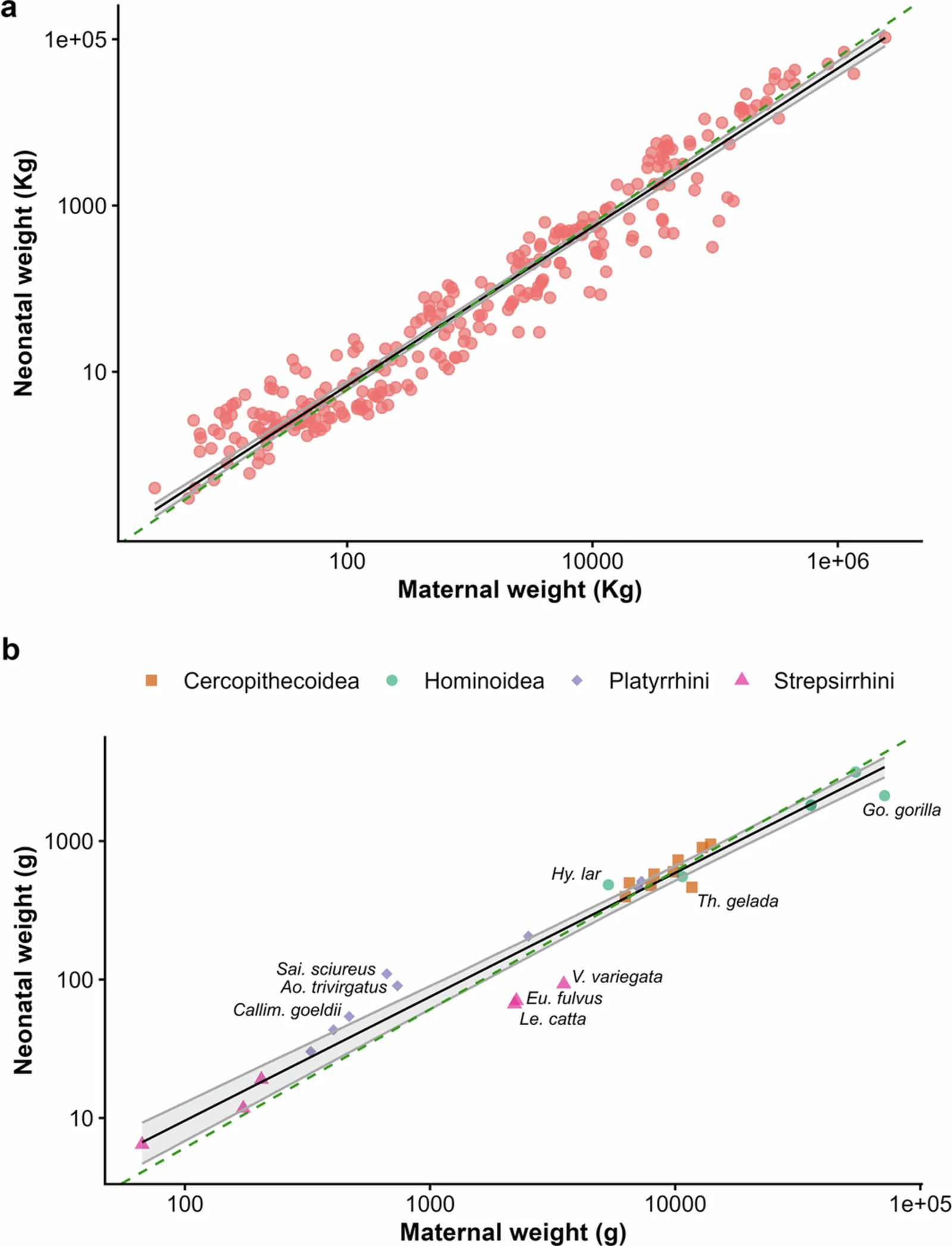
Relationship between neonatal body size and maternal body size across mammals (a) and across primates only (b). **a**, Regression in log scale of neonatal weight (g) against maternal weight (kg) across mammals. The data come from Tague (2016)^58^, except for the primate species included in this study, for which we used the values listed in Supplementary Tables 4 and 5. The black line and shading show the allometric relationship line and 95% confidence interval, estimated through SMA regression (slope = 0.954, 95% CI 0.926 – 0.983, intercept = −1.075, R^2^ = 0.935, p < 0.0001). A one-sample test of the slope (slope.test in package *smatr)* shows that the slope is significantly different from what expected under isometry (isometry slope = 1, p = 0.002), represented by a dashed green line. In other words, there seems to be a pattern of larger species giving birth to relatively smaller babies in respect to maternal size. **b**, Regression in log scale of neonatal weight (g) against maternal weight (kg) across the primate species included in this study. The black line and shading show the allometric relationship line and 95% confidence interval, estimated through SMA regression (slope = 0.895, 95% CI 0.817 – 0.980, intercept = -0.809, R^2^ = 0.947, p < 0.0001). The names of the species falling further from the line are provided. A one-sample test of the slope (slope.test in package *smatr)* shows that the slope is significantly different from what expected under isometry (isometry slope = 1, p = 0.018), represented by a dashed green line, but it is not significantly different from the slope identified for mammals (mammal slope = 0.954, p = 0.160), indicating that larger primates give birth to relatively smaller neonates, but that this allometric relationship is similar in primates to that of the wider mammal taxon.

Across mammals, there is an allometric relationship between maternal and neonatal body size, with smaller species generally giving birth to relatively larger babies^29^. The same allometric pattern exists in primates (Extended Data Fig. 1 and Extended Data Table 1) and could be expected to lead to higher CPP in small-bodies species if relatively larger neonatal bodies are accompanied by similarly larger heads. To the best of our knowledge, however, the relationship between maternal body size and the two key components of CPP, pelvic inlet size and neonatal head size, has never been assessed. Under isometric relationships between neonatal head size, maternal pelvic size and maternal body size, we would expect CPP to be similar across species independently of maternal body size. Instead, we found a strong negative relationship between CPP in face/occiput presentation and maternal body size throughout the clade, with smaller species experiencing a tighter fit than larger ones (slope = −0.247, isometry slope = 0, *P* < 0.0001; Fig. 5 and Table 1). While this is consistent with allometric observations across mammals for neonatal body size^29^, the pattern observed for CPP could be explained by allometry between maternal size and either, or both, of its components.

High CPP could have evolved owing to directional selection towards relatively larger neonates and neonatal heads, toward relatively smaller pelvic canals or even due to a combination of the two. Based on the classic ‘obstetrical dilemma’ hypothesis, humans are considered an example of the latter situation: high CPP emerged as a convergence of a more compact pelvis for effective bipedal locomotion and a larger neonate head due to increased encephalization. In case of isometric change, we would expect pelvic inlet area and neonatal head area to change according to maternal body size; more specifically, as areas are quadratic measures (mm^2^) and maternal weight is a cubic measure (related to volume in mm^3^), under isometry the inlet and head presentation areas are expected to change to a ratio of two-thirds of the maternal weight. To test for allometry, therefore, we need to evaluate whether the slopes of the regression lines between the two elliptical areas and maternal body size are significantly different from two-thirds.

**Fig. 6:**
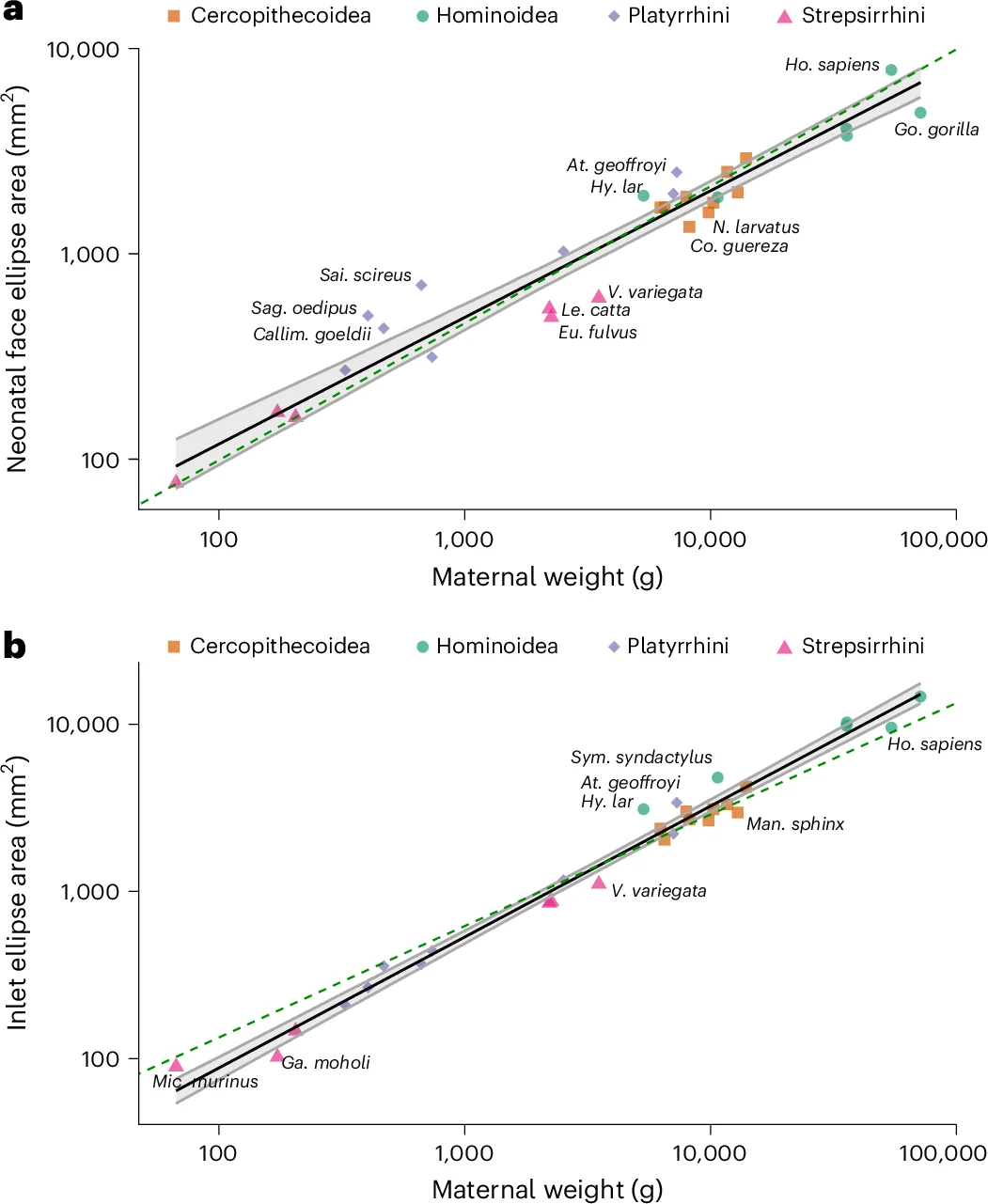
Relationship between neonatal head size and maternal body size and between maternal inlet size and maternal body size across primates. **a**, Regression in log scale of neonatal head size (face/occiput ellipse area) against maternal weight across 29 primate species. The black line and shading show the allometric relationship line and 95% CI, estimated through SMA regression (slope = 0.617, 95% CI 0.555–0.685, intercept = 0.840, *R*^2^ = 0.929, *P* < 0.0001). The slope is not significantly different from what is expected under isometry (isometry slope = 0.667, *P* = 0.139), represented by a dashed green line. In other words, there seems to be a pattern of larger species giving birth to babies with a relatively smaller head with respect to maternal size, but the pattern is not significant. **b**, Regression in log scale of maternal inlet size (ellipse area) against maternal weight across 29 primate species. The black line and shading show the allometric relationship line and 95% CI, estimated through SMA regression (slope = 0.783, 95% CI 0.743–0.826, intercept = 0.378, *R*^2^ = 0.982, *P* < 0.0001). The slope is significantly different from what is expected under isometry (isometry slope = 0.667, *P* < 0.0001), represented by a dashed green line. Smaller species tend to have a relatively smaller pelvic inlet, despite giving birth to relatively bigger neonates. Species that deviate the most from the fitted lines have been labelled.

Our data show a slight, but not significant, allometric pattern of smaller primates giving birth to babies with a relatively larger head (slope = 0.617, isometry slope = 0.667, *P* = 0.139; Fig. 6a), despite the clear allometric pattern identified with neonatal body size. This is probably due to differences in encephalization across primate groups that compensate partially for the allometric trend in fetal head size. The smaller species in our sample are strepsirrhines, characterized by lower encephalization, whereas the larger species are apes, characterized by higher encephalization than other primates^30^. Although we could expect the birth canal to be relatively larger in small species to compensate for their relatively larger neonates and avoid feto–pelvic disproportion, smaller primates tend to have a relatively smaller inlet size (slope = 0.783, isometry slope = 0.667, *P* < 0.0001; Fig. 6b), contributing to increase their CPP (Table 1). Repeating the standardized major axis regression (SMA) using phylogenetic independent contrasts (PICs) returned slopes that were consistent with those from the uncorrected SMA, indicating that the allometric relationships we have identified are robust to phylogenetic structure (Extended Data Table 2).

Together, these results show that allometric changes in pelvic inlet size and, to a less clear extent, fetal head size converge to create higher CPP in smaller species. Unusually high CPP in some species, even with respect to this primate-wide allometric pattern, seem to have emerged in different ways. For humans, the tight fit is due to the convergence of a particularly large headed neonate and an unusually small inlet in comparison to other apes of similar size. The exceptionally high CPP in *Saguinus* (1.87) and *Saimiri* (1.92), seem to be due mostly to a large fetal head in these small-size primates, whereas relatively small neonatal heads explain a low CPP in most lemurs. The tight fit observed in most platyrrhines and in lorises, with CPP above 1.0, is explained by the combined primate-wide allometric trends towards small inlets and large neonatal heads in smaller primates.

## Discussion

Humans have long been considered to display a unique ‘obstetrical dilemma’, arising from the specific conflicting demands of bipedalism and high encephalization^8,9,31^. This interpretation has been often represented and reinforced by the famous diagram based on the work of Schultz^1^, showing a tight cephalopelvic fit in humans with respect to other primates. By using canal measurements better suited to a primate-wide comparison and more realistic fetal measurements, we updated Schultz’s diagram and extended CPP calculations to a much larger number of species, spanning the whole primate order. Our results challenge the past anthropocentric framing and show that humans are neither unique nor unusual in having a tight fit between the neonatal head and the pelvic inlet, with some primate species experiencing even higher obstetric constraints due to allometric patterns leading to smaller pelvic canals and larger neonatal heads in smaller species. Instead of a story of human uniqueness, therefore, we reveal a pattern of many obstetrical dilemmas that reminds us that evolution is often shaped by trade-offs.

### Humans are not the odd ones out

A main message emerging from our study is that research approaches developed on humans are not a good starting point for comparative analyses and can lead to biased interpretations. Our results are consistent with recent work showing that the existing practice of applying human-based anatomical measurements universally overestimates pelvic space in non-human primates^24,25^. This study also supports recent findings that the traditionally defined (human-based) pelvic inlet is not the most restricted level of the canal in non-human primates and that some taxa (for example, *Callithrix*, *Cebus*, *Saimiri*) experience higher CPP than humans. Higher CPP could be mitigated by face-first presentation and pelvic ligament relaxation^11^. In addition, malleability of the newborn head (for example, head moulding) can facilitate its passage through tight passages; although considered unlikely^10,11^, an osteological and histological survey of newborn sutures and fontanelles is yet to be done in a broad range of primates.

Smaller species tend to experience a tighter fit due to two important allometric patterns: they give birth to relatively larger neonates (Extended Data Fig. 1), with often larger heads with respect to maternal body size (different encephalization levels across primate groups confound the allometric pattern; Fig. 6a), while having relatively smaller pelvic inlets (therefore needing to fit a larger neonate through a smaller space; Fig. 6b). The most extreme cases of cephalopelvic disproportion that we have identified occur indeed in small primates, such as bushbabies and squirrel monkeys.

Some species deviate from these general allometric trends, however, suggesting that specific selective pressures might have acted on neonatal or pelvic size. *Saguinus oedipus* and *Saimiri sciureus*, with the highest CPP here, give birth to neonates that are exceptionally large, about 10–15% of maternal body weight^32–34^ and with particularly large fetal heads. It is worth noting that *Saguinus* frequently produces twins; our current dataset does not distinguish between singleton and twin births, and this could affect the interpretation of CPP in callitrichids. Further data would be necessary to account for variation in litter size explicitly. While reliable birth data for *Saguinus* are unavailable, *Saimiri sciureus* can experience neonatal and infant death rates of more than 34%^20^. Such extreme investment in fetal size may reflect evolutionary strategies that favour producing fewer but larger, more precocial offspring with higher long-term survival potential, despite the increased risk of neonate birth injury or death. In these taxa, the pelvic inlet is also relatively small, and the resulting tight cephalopelvic fit may represent a functional trade-off between locomotor requirements favouring a small pelvic canal and neonatal precociality (that is, investment on larger and more developed newborns to improve their survival rate) leading to bigger neonatal bodies and heads, convergently with humans.

Several non-human primate species, including macaques and marmosets, experience obstructed labour. In captivity, caesarean sections are not uncommon^35,36^ to manage delivery complications or ensure neonatal survival. These interventions reflect underlying obstetric challenges, which may be related to relatively high CPP observed in several of these species, although for some species (for example, baboons^37^) neonatal malposition is also an important factor. It is important to consider, however, that captive individuals are often overfed and less active than their wild counterparts, and this may increase the likelihood of complications during birth. In such settings, the risk of feto–pelvic disproportion is sometimes mitigated through caesarean delivery, but it remains unclear how these individuals would fare under natural conditions.

Apes as a group are characterized by low CPP, which can be explained as a direct result of increased maternal body size and the favourable allometry with neonatal (smaller) and inlet (larger) size. Overlaying this pattern, the disproportionally larger inlet in gibbons and siamangs further facilitates neonatal passage. Humans, however, have secondarily evolved a tighter fit than expected for their body size due to both increased neonatal encephalization and a decrease in inlet area, the latter likely reflecting changes in pelvic morphology associated with bipedal walking^38^.

Our observations seem to conflict with recent research reporting a similar cephalopelvic fit in humans and chimpanzees^25^. The apparent contradiction is probably due to the different methodology used, and particularly the way in which CPP was calculated; cephalopelvic fit was estimated by other authors^25^ as the minimum distance between the fetal skull and the maternal inlet when centring the neonatal head within the pelvic bony canal, at a lower level in the canal itself and close to the end of the sacrum^25^. Although the distance between the end of the sacrum and the pubis is smaller at this level than at the inlet, it is unclear what role sacral nutation—the change of inclination of the sacrum during birth—would play in widening the canal passage. In humans, sacral nutation has been estimated to add an additional 1–2 cm^39^ to the anterior–posterior diameter of the outlet—a substantial increase on the average 10 cm diameter—but the effects of nutation are likely to vary among species based on sacral length and whether they have a larger number of fused sacral vertebrae. The inlet area, defined more rigidly by a full circle of bones than the pelvic outlet, is less likely to be affected by sacral nutation and allows a more consistent comparison across species. The conclusion that chimpanzees (and possibly other apes) experience substantial obstetric challenges as a result of a gradual exacerbation of birth constraints across anthropoid primates^25^ is not supported in the present study. Our findings, however, do align with previous research^24^ that likewise reported less tight cephalopelvic fit in chimpanzees (and in the other great apes). The results here indicate that non-human great apes possess a relatively spacious pelvic inlet compared not only to humans^24^, but also to most monkeys^11^.

### Mechanisms to mitigate cephalopelvic disproportions

Several species of primates show a very tight cephalopelvic fit or an extreme disproportion in which the fetal head cannot be accommodated within the modelled inlet geometry, yet successful birth clearly occurs. Therefore, CPP should not be interpreted as a direct proxy for birth difficulty, but rather as a geometric measure of cephalopelvic fit whose functional consequences depend on compensatory mechanisms that facilitate successful birth. Indeed, 281 Japanese macaques monitored for birth across 27 years had no cases of maternal and neonatal mortality that could be linked to a birth event^40^, despite the presence of a relatively high CPP (Figs. 3 and 4). One potential mitigating factor across species is optimizing fetal presentation^25^. Previous research based on maximum fetal cranial breadth and length^1^ implicitly assumed a sinciput presentation in all species. In non-human primates, however, frequent face presentation during birth is well documented, whereas human neonates mostly engage in the canal in occiput anterior presentation^41^. Indeed, in some primates for example, *Saimiri*, *Saguinus* and *Galago*, the disparity between sinciput and face-first presentation is so great that the latter seems to be the only viable option. In baboons, face presentations, characterized by the fetus’s neck being fully extended, are common during delivery^20,37^; similarly, geladas normally display face presentations during birth, with infants maintaining neck extension throughout the process^42^. Face presentation is also well documented in squirrel monkeys^20^ and macaques^43,44^. The findings reported in this study show that face presentation substantially reduces CPP, with the face presenting a smaller area than the head in sinciput position in all species, and may act as an adaptive mechanism to facilitate delivery in species with tighter cephalopelvic fits. The human occiput presentation returns a similar minimized head area, and it is probably more effective in our species due to the difficulty of fully extending the head and aligning it with the spine; due to our erect posture, in fact, the joint between the cranium and the spine is located more anteriorly instead of towards the back of the head.

While our analysis focuses on cephalopelvic fit, it is important to acknowledge that neonatal shoulder breadth can also influence obstetric risk^45^. Shoulder dystocia is a well-recognized cause of obstructed labour in humans^46^, and great apes also present relatively broad shoulders compared with their head width^1^. However, there is currently no comparative dataset on feto–pelvic relationships at the shoulder level that would allow systematic assessment across taxa. Future work integrating fetal shoulder breadth and other dimensions of the neonatal body with pelvic morphology would therefore be essential to fully evaluate the potential contribution of postcranial fetal dimensions in shaping obstetric adaptations.

Additional anatomical features may also mitigate obstetric constraints. Flexibility of the pelvic joints seems to functionally facilitate birth in some mammals^29^. In *Papio* and *Saimiri*, the pelvic ligaments relax and the hipbones dislocate temporarily during labour to increase the inlet area by 30% and 100%, respectively^20^. This loosening of ligaments, together with the presence of an unfused pubic symphysis in some species, may serve to increase the space available during delivery^25,46^. Although pubic bone fusion is widespread among anthropoids, including many platyrrhines (for example, *Saguinus*, *Callithrix*) and cercopithecoids (for example, *Macaca*), it occurs less frequently or not at all in some strepsirrhines; in *Galago moholi*, for example, pubic fusion has not been observed in male or female animals to date, even at post-reproductive age^46^. The open symphysis may therefore represent a derived, obstetrically advantageous trait in small-bodied taxa with relatively large neonates. In some species in which pubic fusion has been observed, it occurs later in female than in male animals (for example *Macaca mulatta* and *Pan troglodytes*), or exclusively in male animals (*Microcebus murinus*)^46^, suggesting an important role in reproduction. In *Macaca mulatta*, most females eventually develop pubic fusion during their reproductive years, indicating that other compensatory mechanisms may be at play^47^. Among hominoids, humans are unique in maintaining an open symphysis throughout life, possibly an adaptation to accommodate particularly large-brained neonates in a more compact pelvis^45^. Female chimpanzees also show delayed or absent pubic fusion until the end of reproductive life, despite their looser cephalopelvic fit^46^. Lack of fusion at the female pubic symphysis seems to have evolved convergently in different lineages, probably under obstetric selection, whereas in others, different mechanisms might mitigate parturition difficulties.

## Conclusion

In this study, we show that Schultz’s influential diagram of cephalopelvic fit in primates, often used to represent the evolutionary outcome of a unique human obstetrical dilemma, is misleading in several respects. By using human standard pelvic measurements, it overestimates and misrepresents the shape of the birth canal inlet in non-human primates. Moreover, it adopts unrealistic neonatal head measurements that do not align with common fetal presentation in humans or other primates. Finally, by limiting the comparison to few primate species, mostly apes, it provides a non-representative view of primate CPP and in particular of human CPP within the order. We demonstrate that a tight cephalopelvic fit is not unique to humans, nor is it restricted to a single clade. Although the human birth canal is tightly matched to neonatal head size, comparable or even tighter fits occur in several non-human primates, including species as distantly related as macaques, squirrel monkeys and bushbabies. This variation is explained partially by allometric patterns, with smaller species giving birth to relatively larger neonates through a relatively smaller pelvic canal. The particularly high CPP observed in some species is associated with exceptionally large neonates, exceptionally small inlets, or a combination of the two, suggesting a range of obstetrical dilemmas across primate species. In turn, these results provide a refined evolutionary context for understanding the diversity of birthing strategies among primates and the comparative context in which human birth difficulties emerged.

## Methods

### Pelvic 3D model collection

This study included a total of 130 adult female specimens of 29 primate species, representing a broad phylogenetic sample of extant primates. Only adult females with fused epiphyses and no visible pelvic pathologies were included in the analyses. Specimen IDs, taxonomical information and source institutions are provided in Supplementary Table 2.

All human data were obtained from existing collections and were fully anonymized before analysis. Access to the data was granted by the respective institutions in accordance with their ethical guidelines and legal requirements, and all necessary permissions for research use were secured. Data from the New Mexico Decedent Image Database (NMDID) were used in accordance with their data use agreement, and we acknowledge: ‘The Free Access Decedent Database funded by the National Institute of Justice grant no. 2016-DN-BX-0144.’ Human CT data from Kyoto University and the Naturhistorisches Museum Wien were used with permission from the respective institutions. Non-human primate data were likewise obtained from curated collections. No animals were euthanized, captured or sedated for the purposes of this study. The CT scans of living animals included in the present study were collected for previous research projects and made available to us by the holding institutions. As this study involves analysis of anonymized archival data and does not involve living human participants or experimental procedures on animals, additional ethical approval was not required.

Pelvic morphology was captured using two complementary 3D data acquisition methods depending on specimen preservation and facilities within the corresponding institution: (1) high-resolution CT scans for osteological and cadaveric specimens, and (2) structured light or surface laser scanning for osteological specimens. CT scans required segmentation of the pelvic bones to isolate them from surrounding soft tissue in the case of cadaveric specimens. Surface-scanned specimens required fusion of several scans taken from different angles to ensure complete surface coverage. All scans were acquired at sufficient resolution to allow accurate anatomical landmarking, with post-processing performed in 3D Slicer v. 5.8.0 to generate clean polygonal meshes in.ply format. In cases where the pelvic bones were preserved as three separate elements (two coxal bones and sacrum), we rearticulated them following a previously published protocol to reconstruct an anatomically connected pelvis^48^.

### Obstetrically constraining birth canal aperture

**Extended Data Fig. 2:**
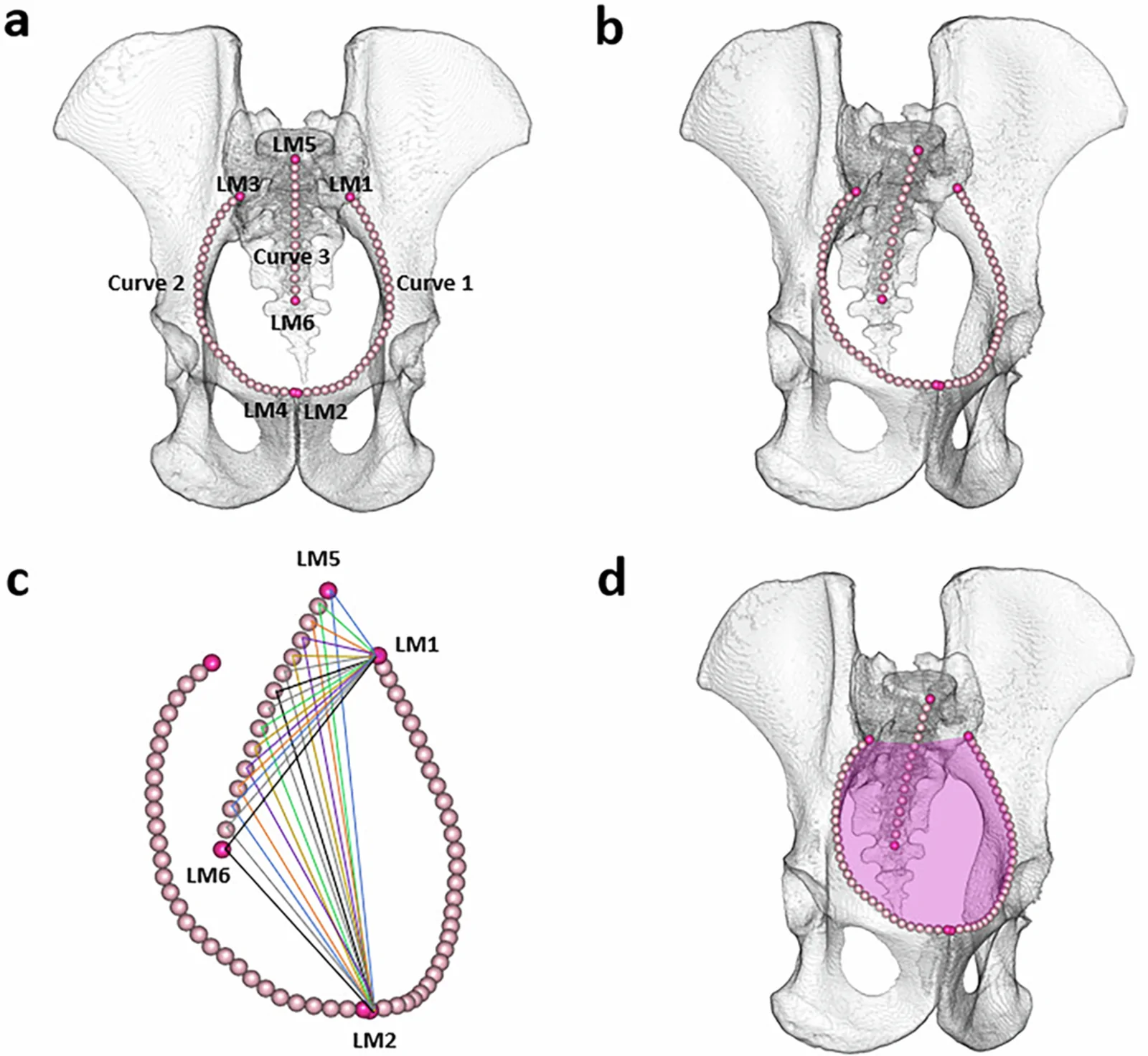
Landmark and semilandmark configuration used to quantify pelvic inlet dimensions, exemplified in *Pan troglodytes*. (**a**) and (**b**) Anterior and anterior-lateral view of a 3D chimpanzee pelvis showing the three anatomical curves used to capture pelvic aperture shape and dimensions: the left and right inlet curves and the anterior sacral curve. The landmark configuration shows two fixed landmarks and 28 semilandmarks along each inlet curve (left and right), two fixed landmarks and 13 semilandmarks along the anterior sacral curve, and two fixed landmarks at the top of the pubic symphysis (left and right). (**c**) Diagram showing the computation of the most obstetrically relevant inlet dimension, obtained by summing the linear distance between LM1 and the relevant sacral landmark with the distances between the relevant sacral landmark and LM2. (**d**) 2D inlet plane (pink) derived from the combined landmark configuration corresponding to the minimum summed distance.

For each individual 3D pelvic model, we used 3D Slicer v. 5.8.0 to place anatomical landmarks and semi-landmarks along three curves (Extended Data Fig. 2 and Extended Data Table 3): (1) the left and right pelvic inlet margins along the iliopectineal line (each with 28 semi-landmarks spaced equally between two fixed landmarks) and (2) the anterior sagittal curve of the sacrum (13 semi-landmarks enclosed by two fixed landmarks). Instead of relying on the traditional human-based definition to identify the obstetrically constraining inlet plane in non-human primates we combined the landmarks and semi-landmarks along the iliopectineal line with the central landmarks at the top of the posterior superior pubic symphysis, and a point on the sacrum that minimized the anterior–posterior diameter of the inlet while lying close to the plane formed by the other inlet landmarks (Extended Data Fig. 2). The identification of this sacral landmark is complicated by the variation in the shape and position of the sacrum across primates. Whereas in humans the central anterior margin of the promontorium lies close to the inlet brim and constrains the fetal passage, in other primates the higher location of the sacrum with respect to the pubis and the generally less prominent promontorium mean that a lower part of the sacrum serves to delimit the inlet^20^. To identify this sacral landmark in other primates, for each individual we minimized the sum of two distances: (1) from a given landmark/semi-landmark along the anterior sacral curve to a fixed landmark at the intersection of the iliopectineal line with the auricular surface (landmark 1), and (2) from the same sacral landmark/semi-landmark to a fixed landmark located at the top of the left pubic symphysis (landmark 2), therefore measuring the anterior–posterior diameter. These sums were computed for each sacral semi-landmark and averaged within species. The sacral landmark associated with the smallest sum of these two distances (Extended Data Table 4) was used to complete the constraining inlet plane. The rationale for this method is to identify a position on the sacrum that lies not too far from the other inlet landmarks (it lies more or less on the same plane and therefore would contribute to constrain the head passage as it navigates that level of the canal) while also minimizing the sagittal inlet diameter. This method accounts for individual and species variation in canal and sacral morphology.

Once the landmarks and semi-landmarks enclosed the obstetrically constraining inlet area were determined, these were projected on a two-dimensional (2D) plane using a principal component analysis (PCA) computed from the 3D coordinates. The resulting PC-scores are the coordinates rotated in a way that the first two dimensions cover the main 2D variation and the third dimension the shallow depth in 3D. By removing the third dimension and only using the coordinates of the first two PCs, a 2D projection of the 3D inlet is obtained.

### Neonatal head and face dimensions

We collected three cranial measurements of 40 neonates from the same 29 species for which maternal pelvic data were available. Neonatal stage was determined from specimen records (0–10 days old) and by inspection of physical characteristics according to previous literature^49^. The measurements collected were: (1) occipitofrontal diameter, measured from the most posterior point of the occiput to the root of the nose (nasion), used to approximate head length in sinciput presentation; (2) submento–bregmatic diameter, measured from the middle point between the angles of the mandible (gnathion–gnathion) to bregma, representing head height in face presentation; and (3) biparietal diameter, measured across the widest transverse diameter of the cranium at the level of the parietal eminences, representing cranial breadth in face presentation. These cranial measurements are based on ref. ^2^

**Extended Data Fig. 3:**
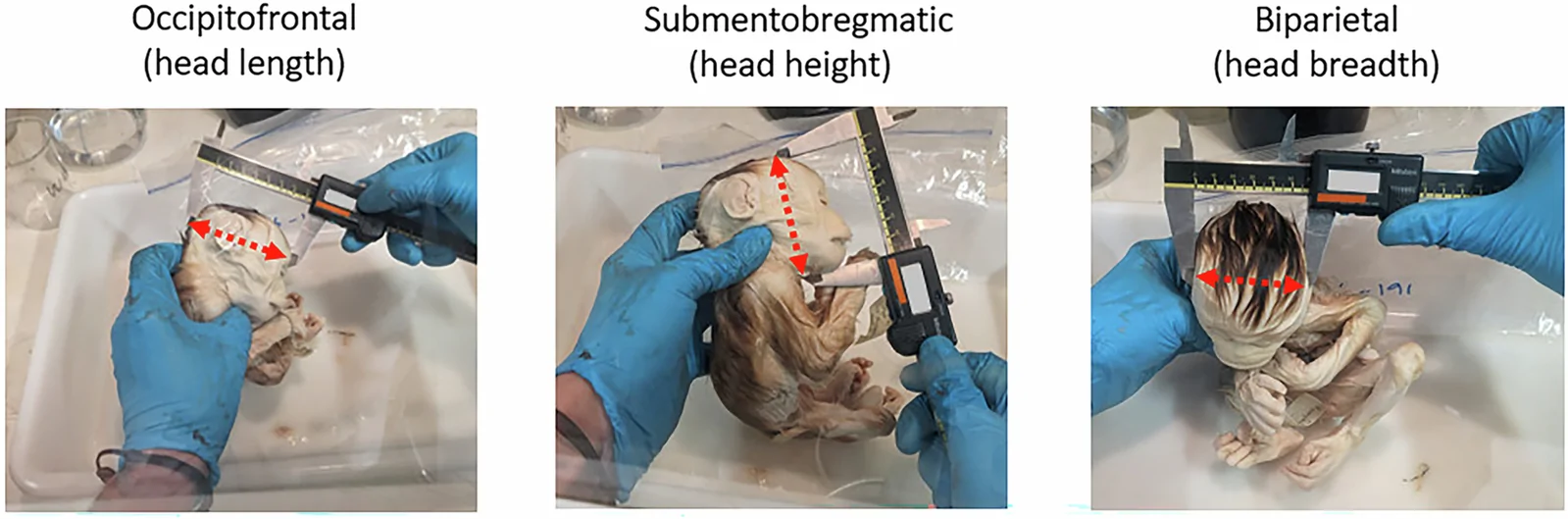
Neonatal cranial measurements used to construct birth-relevant head and face ellipses. Three linear cranial dimensions were collected directly from preserved neonatal primates using digital calipers, or from 3D models of neonate cadavers using Meshlab v.2023.12 software. These measurements were used to model fetal head size and shape in relation to maternal pelvic dimensions. All measurements were taken by the same researcher to ensure consistency.

Depending on the condition in which the specimen was made available to us, measurements were obtained either directly from formalin-preserved physical specimens using a digital caliper (Extended Data Fig. 3), from 3D models generated from CT scans of formalin-preserved or frozen specimens using the Meshlab v. 2025-07, or from published measurements. When data were available from different literature sources^1,2,43,50^, we calculated the species value by averaging all measurements. In the case where values in the literature already represented species averages, we combined them with any additional measurements by computing a weighted mean that took into account the sample size of the published averages.

Detailed information on each individual, the cranial diameters measured, and their source (either hosting institution or literature) are provided in Supplementary Tables 2 and 3.

### Cephalopelvic proportions

For each species, we obtained a representative traditional inlet shape (based on standard human landmarks) and an obstetrically constraining inlet shape by (1) superimposing the landmarks for all individuals using a partial Procrustes superimposition that preserved differences in scale but removed differences in location and rotation; (2) extracting the mean inlet shape. For humans, the standard inlet definition was used as the obstetrically constraining one. To find the area of the inlet that realistically could be filled by the neonate’s head, we inscribed the maximum ellipse into the traditional inlet and constraining inlet polygons, using a newly developed function that allows the ellipse to rotate freely so it can optimally fit the available space enclosed by the 2D projection of the 3D inlet coordinates. As there exists no closed form solution to this problem, we approached this issue using an iterative algorithm that we implemented in the R package ‘Morpho’ (function ‘inscribeEllipseRot’)^51^. It starts with a reasonable position (ellipse centred to the centroid of the 2D projection of the birth canal) and iteratively (999 iterations) aims to find the largest ellipse to be fitted into the birth canal parametrization. To account for rotational optimization, the algorithm runs all 999 iterations over a stepwise rotation using 2° steps. The largest inscribed ellipses defined the shape and area available for the passage of the fetus in each species. Maternal inlet area was, therefore, calculated for the standard and the obstetrically constraining inlet plane based on the area of the inscribed ellipses rather than on the raw projected 2D inlet polygons (Fig. 2). We compared the ellipse areas for the traditional and constraining inlets using a paired *t*-test, to evaluate whether our definition of the obstetrically constraining pelvic inlet returned a significantly different estimation of the space available for the passage of the neonate.

We then constructed ellipses representing the neonatal head using the species averages of the collected measurements. For sinciput presentation, the radii of the ellipse were calculated as half of the occipitofrontal length and half of the biparietal breadth; for face presentation, we used half of the submento–bregmatic height (suboccipito–bregmatic for humans) and half of the biparietal breadth. The fetal and pelvic ellipses were superimposed within the same plane by translating them to the same ellipse centroid, allowing direct visual comparison of inlet shape and head dimensions.

CPP was defined as the ratio of the fetal head area (modelled as an ellipse) to the maternal pelvic inlet area (maximum inscribed ellipse), separately for sinciput and face/occiput presentation. We tested for allometry in the relationship between CPP and maternal body size, given the central role of the latter in influencing both neonatal and pelvic dimensions, as well as the relationship between maternal body size and inlet size, and the size of the neonatal body and head. Data on neonatal bodyweight were collated from refs. ^34,52^ and data on maternal bodyweight were collated from refs. ^34,53^ and are provided in Supplementary Tables 4 and 5.

We used SMA, using the R package ‘smatr’^54^, to identify the line that best captures the relationship between maternal body size and the other variables; all variables were transformed using the base 10 logarithm to facilitate comparison across measures that change on different scales and orders of magnitude, and because we are interested in proportional rather than absolute variation. We tested for difference between the slope of the relationship lines with the predicted slope under isometry. The theoretical isometric slope depended on the variables analysed and whether they changed on a linear, quadratic or cubic scale. The isometric slope is calculated as the ‘dimensional exponent’ of variable *y* over the one of *x*. For example, the isometric slope of the inlet area (dimensional exponent = 2) against maternal weight (dimensional exponent = 3) is two-thirds or 0.667. As CPP is a ratio, the dimensional exponent is calculated by subtracting the exponent of the numerator from the one of the denominator. CPP was calculated as area face/area inlet, hence the dimensional exponent of CPP was 2 − 2 = 0. The isometric slope against maternal body size was therefore 0/3 = 0. In other words, a flat horizontal line of no expected relationship between CPP and maternal weight under isometry.

For the purpose of our study, in which we consider the different variables that could contribute to high CPP and evaluate the functional scaling constraints operating across living primate species, the among-species differences are biologically meaningful independently of phylogenetic patterns of variation. Nonetheless, to confirm that shared ancestry does not substantially distort the estimated scaling slopes, all regressions were repeated using PICs^55^ computed on log-transformed variables and forced through the origin. Where PIC slopes were consistent with those from the uncorrected SMA, the allometric relationships were considered robust to phylogenetic structure.

All statistical analyses were conducted in R v. 4.5.1 (ref. ^56^), and all data and codes are openly available (‘Data Availability’ and ‘Code Availability’).

### Reporting summary

Further information on research design is available in the Nature Portfolio Reporting Summary linked to this article.

## Supplementary information

Reporting Summary

Supplementary Tables 1–5 Supplementary Tables 1–5.

## Figures and Tables

**Extended Data Table 1:**
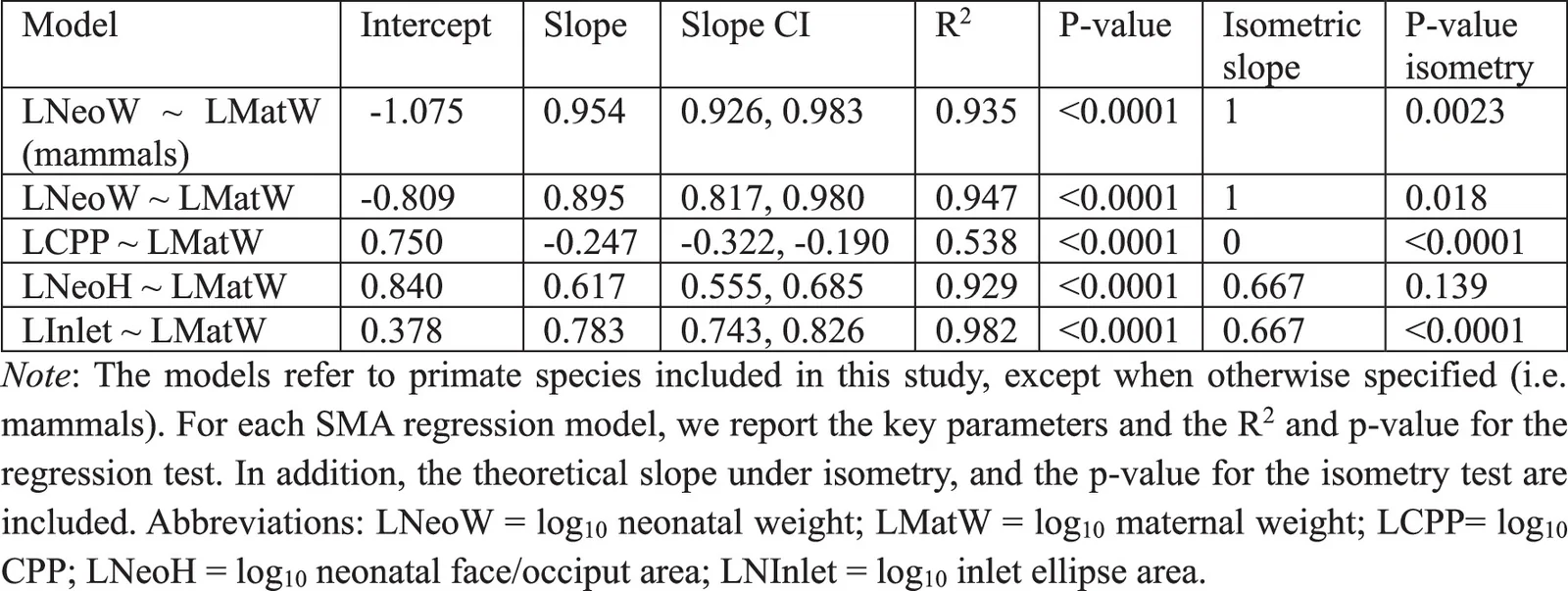
Summary of results for the allometry models

**Extended Data Table 2:**
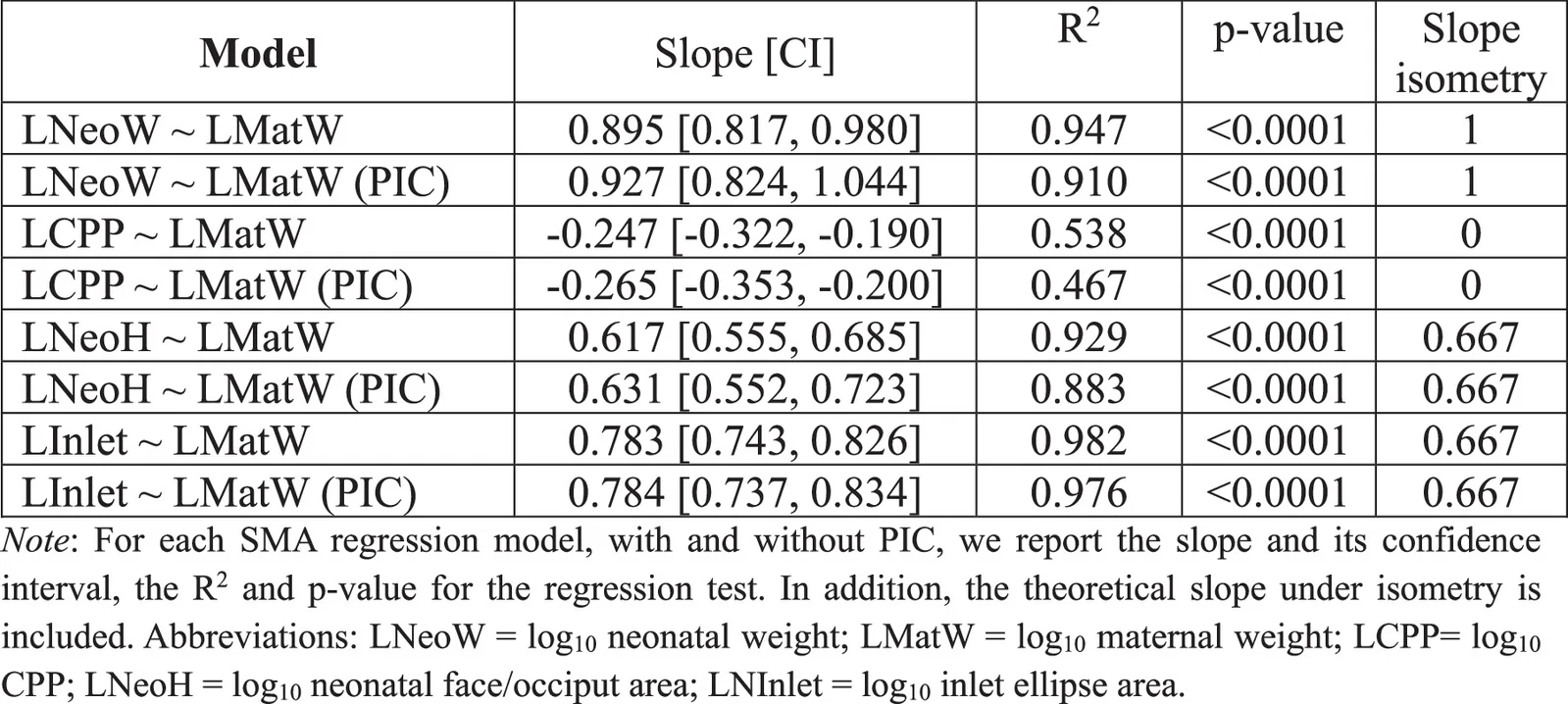
Comparison of key results of the primate SMA models with and without PIC

**Extended Data Table 3:**
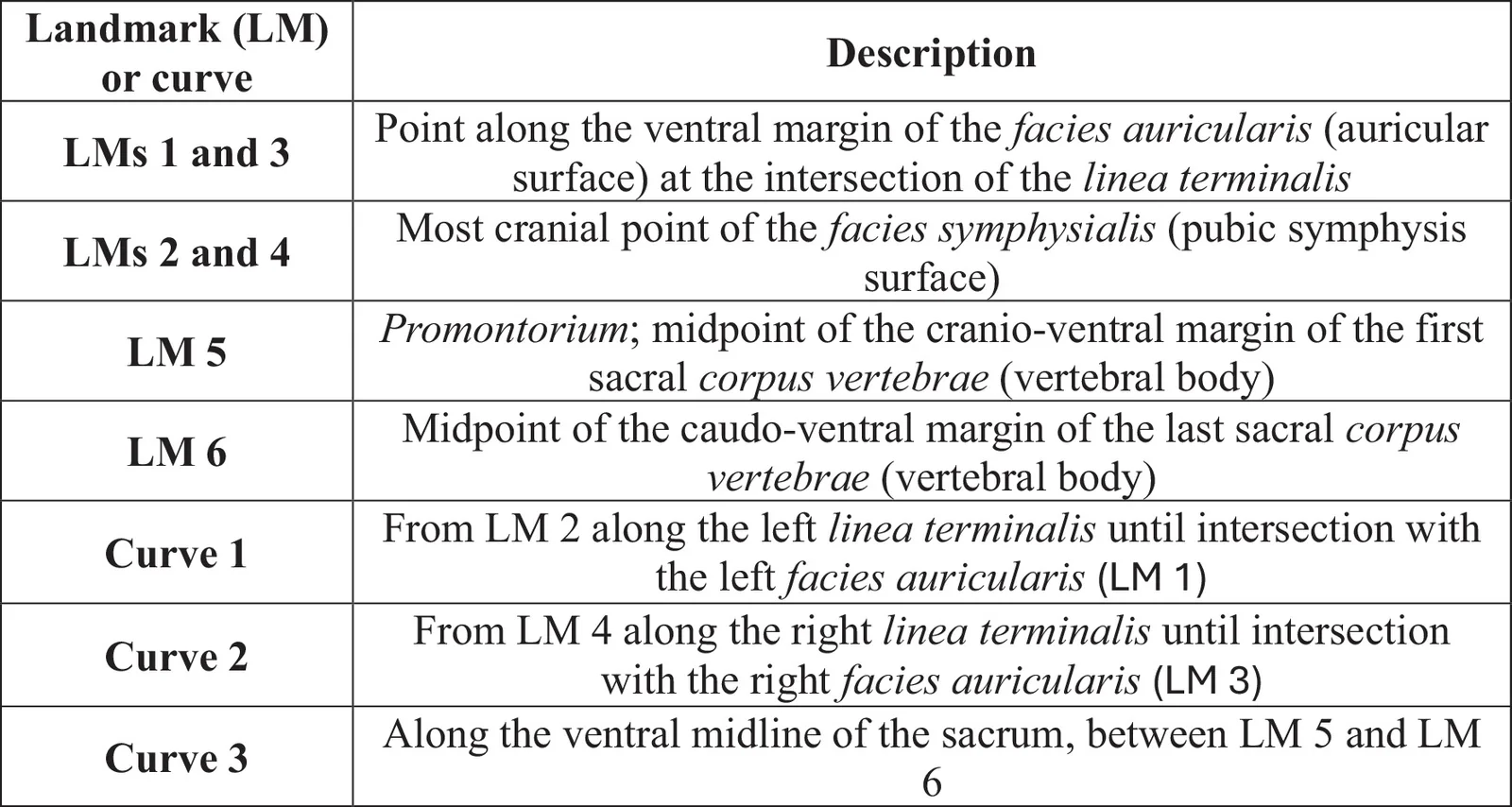
Definitions of anatomical landmarks and curves

**Extended Data Table 4:**
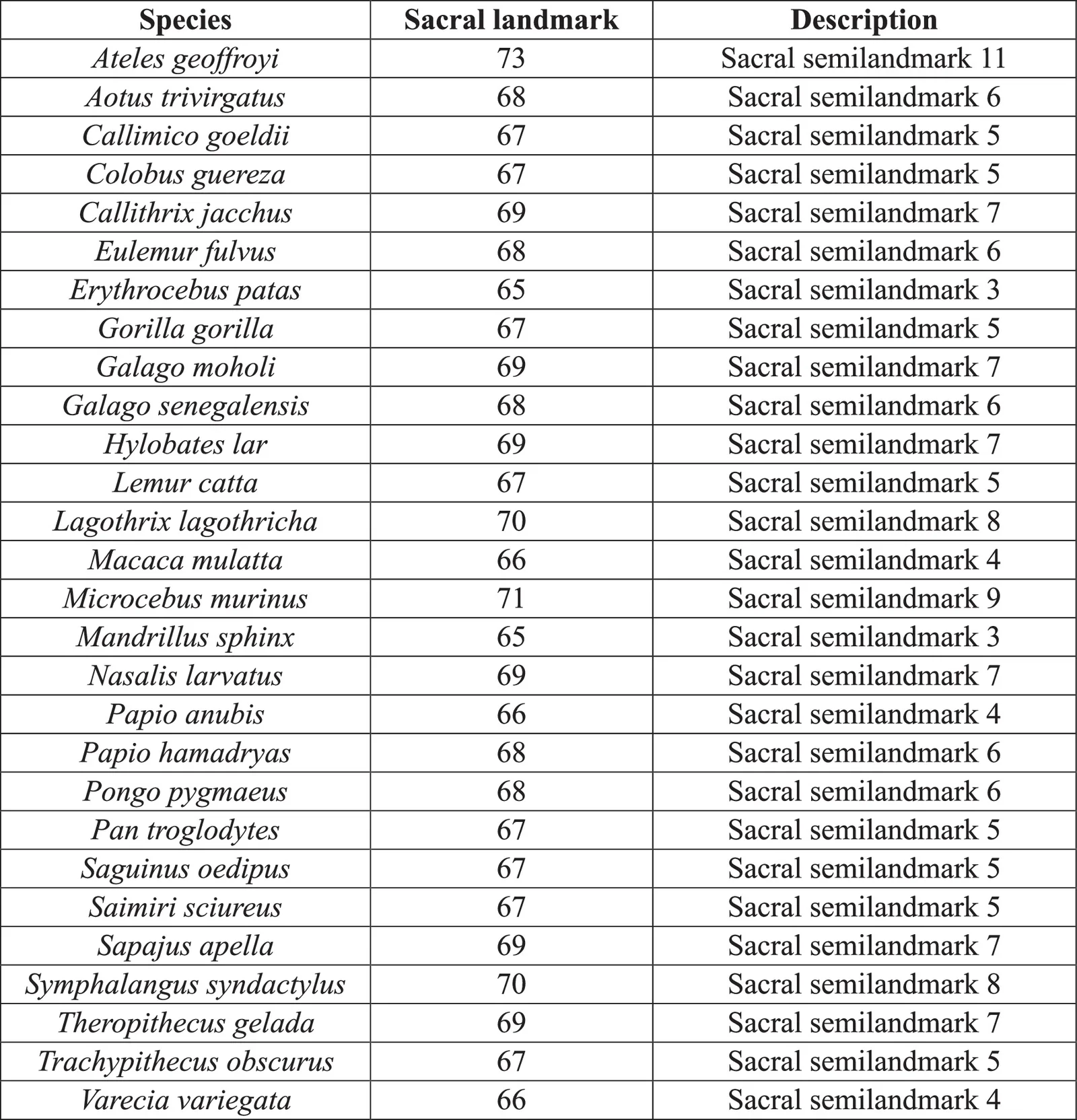
Sacral landmark selected per species that returns the lowest summed distance

## Data Availability

The raw data supporting the findings of this study consist of CT scans of human and non-human primate skeletal remains curated in institutional collections. Owing to institutional and legal restrictions, these data are not publicly available. Human and non-human datasets can be accessed with permission from the respective holding institutions provided in Supplementary Table 2. All other data supporting the finding of this study are available in the Supplementary Information and in Open Science Framework (https://osf.io/2yk8s/).

## Code availability

The code (R scripts) to replicate the analyses is available in the Supplementary Information and in Open Science Framework (https://osf.io/2yk8s/).

## References

[1] Schultz, A. H. Sex differences in the pelves of primates. *Am. J. Phys. Anthropol.* **7**, 401–423 (1949). 15392785 10.1002/ajpa.1330070307

[2] Leutenegger, W. Beziehungen zwischen der Neugeborenengrösse und dem Sexualdimorphismus am Becken bei simischen Primaten. *Folia Primatol.* **12**, 224–235 (1970). 10.1159/000155292 5417922

[3] Leutenegger, W. Functional aspects of pelvic morphology in simian primates. *J. Hum. Evol.* **3**, 207–222 (1974).

[4] Lindburg, D. G. Primate obstetrics: the biology of birth. *Am. J. Primatol.* **3**, 193–199 (1982).

[5] Gruss, L. T. & Schmitt, D. The evolution of the human pelvis: changing adaptations to bipedalism, obstetrics and thermoregulation. *Philos. Trans. R. Soc. Lond. B Biol. Sci.* **370**, 20140063 (2015). 25602067 10.1098/rstb.2014.0063 PMC4305164

[6] Trevathan, W. R. *Human Birth: an Evolutionary Perspective* (Routledge, 2017).

[7] Trevathan, W. Primate pelvic anatomy and implications for birth. *Philos. Trans. R. Soc. Lond. B Biol. Sci.* **370**, 20140065 (2015). 25602069 10.1098/rstb.2014.0065 PMC4305166

[8] Washburn, S. L. Tools and human evolution. *Sci. Am.* **203**, 63–75 (1960). 13843002

[9] Rosenberg, K. R. The evolution of modern human childbirth. *Yearb. Phys. Anthropol.* **35**, 89–124 (1992).

[10] Haeusler, M. et al. The obstetrical dilemma hypothesis: there’s life in the old dog yet. *Biol. Rev. Camb. Philos. Soc.* **96**, 2031–2057 (2021). 34013651 10.1111/brv.12744 PMC8518115

[11] Laudicina, N. M., Piasecki, E., Stoller, M. & Cartmill, M. Obstetric constraints in six monkey genera. *Am. J. Biol. Anthropol.* **187**, e70092 (2025). 40616436 10.1002/ajpa.70092

[12] Saiyed, S. T., Liubicich, R. C., Fidino, M. & Ross, S. R. Stillbirth rates across three ape species in accredited American zoos. *Am. J. Primatol.* **80**, e22870 (2018). 29756650 10.1002/ajp.22870

[13] Abee, C. R. The squirrel monkey in biomedical research. *ILAR J.* **31**, 11–20 (1989).

[14] Debyser, L. W. J. Platyrrhine juvenile mortality in captivity and in the wild. *Int. J. Primatol.* **16**, 909–933 (1995).

[15] Bowden, D., Winter, P. & Ploog, D. Pregnancy and delivery behavior in the squirrel monkey (*Saimiri sciureus*) and other primates. *Folia Primatol.* **5**, 1–42 (1967). 10.1159/000161936 4961868

[16] Sesbuppha, W. et al. Stillbirths in *Macaca fascicularis*. *J. Med. Primatol.* **37**, 169–172 (2008). 18194223 10.1111/j.1600-0684.2007.00275.x PMC2574872

[17] Roura-Torres, B., Amblard-Rambert, P., Lepou, P., Kappeler, P. M. & Charpentier, M. J. E. Stillbirth of a mandrill (*Mandrillus sphinx*) in the wild: perinatal behaviors and delivery sequences. *Primates* **65**, 75–80 (2024). 38133716 10.1007/s10329-023-01112-6 PMC10884356

[18] Schlabritz-Loutsevitch, N. E. et al. The baboon model (*Papio hamadryas*) of fetal loss: maternal weight, age, reproductive history and pregnancy outcome. *J. Med. Primatol.* **37**, 337–345 (2008). 19017195 10.1111/j.1600-0684.2008.00297.x PMC3016714

[19] Pillay, K. R. & Downs, C. T. Pregnancy complications in wild vervet monkeys in an urban mosaic landscape. *Afr. J. Ecol.* **62**, e13251 (2024).

[20] Stoller, M. K. *The Obstetric Pelvis and Mechanism of Labor in Non-human Primates*. PhD thesis, Univ. Chicago (1995).

[21] Demuru, E., Ferrari, P. F. & Palagi, E. Is birth attendance a uniquely human feature? New evidence suggests that bonobo females protect and support the parturient. *Evol. Hum. Behav.* **39**, 502–510 (2018).

[22] Pan, W. et al. Birth intervention and non-maternal infant-handling during parturition in a nonhuman primate. *Primates* **55**, 483–488 (2014). 24859849 10.1007/s10329-014-0427-1

[23] Abitbol, M. M. Ontogeny and evolution of pelvic diameters in anthropoid primates and in *Australopithecus afarensis* (AL 288-1). *Am. J. Phys. Anthropol.* **85**, 135–148 (1991). 1909098 10.1002/ajpa.1330850203

[24] Laudicina, N. M. & Cartmill, M. Bony birth-canal dimensions and obstetric constraints in hominoids. *Am. J. Biol. Anthropol.* **180**, 442–452 (2022).

[25] Webb, N. M. et al. Gradual exacerbation of obstetric constraints during hominoid evolution implied by re-evaluation of cephalopelvic fit in chimpanzees. *Nat. Ecol. Evol.* **8**, 2228–2238 (2024). 39443698 10.1038/s41559-024-02558-7

[26] Hammond, A. S. & Almecija, S. Lower ilium evolution in apes and hominins. *Anat. Rec.* **300**, 828–844 (2017). 10.1002/ar.23545 28406561

[27] Senevirathne, G. et al. The evolution of hominin bipedalism in two steps. *Nature* **645**, 952–963 (2025). 40866708 10.1038/s41586-025-09399-9 PMC12460174

[28] Gherman, R. B., Tramont, J., Muffley, P. & Goodwin, T. M. Analysis of McRoberts’ maneuver by x-ray pelvimetry. *Obstet. Gynecol.* **95**, 43–47 (2000). 10636500 10.1016/s0029-7844(99)00445-7

[29] Grunstra, N. D. S. et al. Humans as inverted bats: a comparative approach to the obstetric conundrum. *Am. J. Hum. Biol.* **31**, e23227 (2019). 30810261 10.1002/ajhb.23227 PMC6492174

[30] Alba, D. M. Cognitive inferences in fossil apes (Primates, Hominoidea): does encephalization reflect intelligence? *J. Anthropol. Sci.* **88**, 11–48 (2010). 20834049

[31] Rosenberg, K. R. & Trevathan, W. R. Birth, obstetrics and human evolution. *BJOG* **109**, 1199–1206 (2002). 12452455 10.1046/j.1471-0528.2002.00010.x

[32] Garber, P. A. & Leigh, S. R. Ontogenetic variation in small-bodied New World primates: implications for patterns of reproduction and infant care. *Folia Primatol.* **68**, 1–22 (1997). 10.1159/000157226 9170641

[33] Smith, R. J. & Jungers, W. L. Body mass in comparative primatology. *J. Hum. Evol.* **32**, 523–559 (1997). 9210017 10.1006/jhev.1996.0122

[34] Smith, R. J. & Leigh, S. R. Sexual dimorphism in primate neonatal body mass. *J. Hum. Evol.* **34**, 173–201 (1998). 9503093 10.1006/jhev.1997.0190

[35] Prestes, N. C. et al. Cesarean sections in marmosets: white-tufted marmoset (*Callithrix jacchus*). *Vet. Zootec.* **21**, 92–97 (2014).

[36] Mosdol, G. Caesarean section in the Java monkey (*Macaca irus*): a case report. *J. Small Anim. Pract.* **17**, 519–525 (1976). 823378 10.1111/j.1748-5827.1976.tb06995.x

[37] Schlabritz-Loutsevitch, N. et al. Parturition in baboons (*Papio* spp.). *Sci. Rep.* **8**, 1174 (2018). 29352119 10.1038/s41598-018-19221-4 PMC5775344

[38] Lovejoy, C. O. Evolution of human walking. *Sci. Am.* **259**, 118–125 (1988). 3212438 10.1038/scientificamerican1188-118

[39] Borell, U. & Fernström, I. The movements at the sacro-iliac joints and their importance to changes in the pelvic dimensions during parturition. *Acta Obstet. Gynecol. Scand.* **36**, 42–57 (1957). 13424209 10.3109/00016345709158023

[40] Pink, K. E. et al. No birth-associated maternal mortality in Japanese macaques (*Macaca fuscata*) despite giving birth to large-headed neonates. *Proc. Natl Acad. Sci. USA* **121**, e2316189121 (2024). 39374390 10.1073/pnas.2316189121 PMC11494302

[41] Arulkumaran, S., Ledger, W., Denny, L. & Doumouchtsis, S. *Oxford Textbook of Obstetrics and Gynaecology* (Oxford Univ. Press, 2019).

[42] Nguyen, N. et al. Comparative primate obstetrics: observations of 15 diurnal births in wild gelada monkeys (*Theropithecus gelada*) and their implications for understanding human and nonhuman primate birth evolution. *Am. J. Phys. Anthropol.* **163**, 14–29 (2017). 28144947 10.1002/ajpa.23141

[43] Kawada, M., Nakatsukasa, M., Nishimura, T., Kaneko, A. & Morimoto, N. Covariation of fetal skull and maternal pelvis during the perinatal period in rhesus macaques and evolution of childbirth in primates. *Proc. Natl Acad. Sci. USA* **117**, 21251–21257 (2020). 32817513 10.1073/pnas.2002112117 PMC7474677

[44] Duboscq, J., Neumann, C., Perwitasari-Farajallah, D. & Engelhardt, A. Daytime birth of a baby crested black macaque (*Macaca nigra*) in the wild. *Behav. Processes* **79**, 81–84 (2008). 18565691 10.1016/j.beproc.2008.04.010

[45] Smelzer, J. S. Prevention and management of shoulder dystocia. *Clin. Obstet. Gynecol.* **29**, 299–308 (1986). 3720062 10.1097/00003081-198606000-00011

[46] Torres-Tamayo, N., Buck, L. T., Hirasaki, E., Rae, T. C. & Betti, L. Variation in pubic symphysis fusion across primates: implications for obstetric adaptation. *Am. J. Biol. Anthropol.* **186**, e25064 (2025). 39910857 10.1002/ajpa.25064 PMC11799747

[47] Morimoto, N., Kawada, M., Tomizawa, Y., Kaneko, A. & Nishimura, T. Pelvic shape change in adult Japanese macaques and implications for childbirth at old age. *Proc. Natl Acad. Sci. USA* **120**, e2300714120 (2023). 37459534 10.1073/pnas.2300714120 PMC10372569

[48] Torres-Tamayo, N., Rae, T. C., Hirasaki, E. & Betti, L. Testing the reliability of the rearticulation of osteological primate pelves in comparative morphological studies. *Anat. Rec.* **307**, 2816–2833 (2024). 10.1002/ar.25366 38112056

[49] Smith, T. D., DeLeon, V. B., Vinyard, C. & Young, J. W. *Skeletal Anatomy of the Newborn Primate* (Cambridge Univ. Press, 2020).

[50] Hamada, Y., Udono, T., Teramoto, M. & Hayasaka, I. Body, head, and facial growth: comparison between macaques (*Macaca fuscata*) and chimpanzee (*Pan troglodytes*) based on somatometry. *Ann. Anat.* **186**, 451–461 (2004). 15646278 10.1016/S0940-9602(04)80082-7

[51] Schlager, S. In *Statistical Shape and Deformation Analysis* (ed. Zheng, G.) 217–256 (Elsevier, 2017).

[52] Kappeler, P. M. & Pereira, M. E. *Primate Life Histories and Socioecology* (Univ. Chicago Press, 2003).

[53] Leutenegger, W. Maternal–fetal weight relationships in primates. *Folia Primatol* **20**, 280–293 (1973). 10.1159/000155580 4208250

[54] Warton, D. I. & Weber, N. C. Common slope tests for bivariate errors-in-variables models. *Biometrical J.* **44**, 161–174 (2002).

[55] Felsenstein, J. Phylogenies and the comparative method. *Am. Nat.* **125**, 1–15 (1985).

[56] R Core Team. *R: A Language and Environment for Statistical Computing* (R Foundation for Statistical Computing, 2024).

[57] Rosenberg, K. R. & Trevathan, W. R. Bipedalism and human birth: the obstetrical dilemma revisited. *Evol. Anthropol.* **4**, 161–168 (1995).

[58] Tague, R. G. Pelvic sexual dimorphism among species monomorphic in body size: relationship to relative newborn body mass. *J. Mammal.* **97**, 503–517 (2016). 30302031 10.1093/jmammal/gyv195 PMC6169469

